# Polymorphism patterns in Duffy-binding protein among Thai *Plasmodium vivax *isolates

**DOI:** 10.1186/1475-2875-7-112

**Published:** 2008-06-26

**Authors:** Panita Gosi, Srisin Khusmith, Thareerat Khalambaheti, David E Lanar, Kurt E Schaecher, Mark M Fukuda, Scott R Miller

**Affiliations:** 1 Department of Microbiology and Immunology, Faculty of Tropical Medicine, Mahidol University, 420/6 Rajvithi Road, Bangkok 10400, Thailand; 2 Department of Immunology and Medicine, Armed Forces Research Institute of Medical Science-United States Army MilitaryComponent, Bangkok, Thailand; 3 Walter Reed Army Institute forResearch, Washington, District of Columbia, USA

## Abstract

**Background:**

The Duffy-binding protein II of *Plasmodium vivax *(*PvDBPII*) has been considered as an attractive target for vaccine-mediated immunity despite a possible highly polymorphic nature. Among seven *PvDBP *domains, domain II has been shown to exhibit a high rate of nonsynonymous polymorphism, which has been suggested to be a potential immune (antibody binding) evasion mechanism. This study aimed to determine the extent of genetic polymorphisms and positive natural selection at domain II of the *PvDBP *gene among a sampling of Thai *P. vivax *isolates.

**Methods:**

The *PvDBPII *gene was PCR amplified and the patterns of polymorphisms were characterized from 30 Thai *P. vivax *isolates using DNA cloning and sequencing. Phylogenetic analysis of the sequences and positive selection were done using DnaSP ver 4.0 and MEGA ver 4.0 packages.

**Results:**

This study demonstrated a high rate of nonsynonymous polymorphism. Using Sal I as the reference strain, a total of 30 point-mutations were observed in the *PvDBPII *gene among the set of Thai *P. vivax *isolates, of which 25 nonsynonymous and five synonymous were found. The highest frequency of polymorphism was found in five variant amino acids (residues D384G, R390H, L424I, W437R, I503K) with the variant L424I having the highest frequency. The difference between the rates of nonsynonymous and synonymous mutations estimated by the Nei and Gojobori's method suggested that *PvDBPII *antigen appears to be under selective pressure. Phylogenetic analysis of *PvDBPII *Thai *P. vivax *isolates to others found internationally demonstrated six distinct allele groups. Allele groups 4 and 6 were unique to Thailand.

**Conclusion:**

Polymorphisms within *PvDBPII *indicated that Thai vivax malaria parasites are genetically diverse. Phylogenetic analysis of DNA sequences using the Neighbour-Joining method demonstrated that Thai isolates shared distinct alleles with *P. vivax *isolates from different geographical areas. The study reported here will be valuable for the development of *PvDBPII*-based malaria vaccine.

## Background

Molecular mechanisms of invasion by *Plasmodium vivax *merozoites are mediated by the Duffy-binding-like (DBL) family of homologous erythrocyte binding protein (EBPs) located within the micronemes of merozoites that recognize specific receptors on red blood cell [1]. *PvDBP *is a 140-kDa protein belonging to a family of erythrocyte binding proteins characterized by a functionally conserved cysteine-rich region. The similarity among DBL families of EBPs is the greatest within two adjacent cysteine-rich domains, designated as the amino cysteine-rich domain (N-cys) and carboxyl cysteine-rich domain (C-cys) [2]. The amino cysteine-rich domain has been identified as the principle adhesion region binding to erythrocyte receptors, while the function of the carboxyl cysteine-rich domain remains unclear. This amino-cysteine-rich domain is present within the 330 amino acid region II, which has been shown to contain the binding motifs necessary for adherence to the Duffy antigen receptor for chemokines (DARC), required for erythrocyte invasion [3]. Critical binding motifs in *PvDBPII *have been mapped to a 170 amino acid spanning region between cysteines 4 and 7 (amino acids 291–460) [4]. Cysteine residues are conserved within the identified binding motif, whereas other amino acids are highly polymorphic, having a high ratio of nonsynonymous to synonymous mutations [5].

The polymorphic nature of the *PvDBP*, particularly its region II (*DBPII*) is a major impediment to the development of a broadly protective vaccine against vivax malaria. A study in Papua New Guinea showed 43 and 18 unique nonsynonymous mutations in 10 Madang [6] and 24 Wosera vivax isolates [7], respectively, whereas 19 unique nonsynonymous mutations were described in 10 Colombian vivax isolates [8]. Often the mutations were found in corresponding locations in the protein sequence of isolates from different geographical areas. Amino acids substitutions at D384G, K386(N/Q), N417K, L424I, W437R, and I503K were found in both Papua New Guinea isolates and Colombia isolates [5-8]. Such results demonstrate that many similar alleles are widely distributed among *P. vivax *from different geographical areas. Polymorphic patterns in the *PvDBP *cluster within the critical erythrocyte-binding segment imply the existence of a selection pressure on *PvDBP *[9]. Under the influence of immune pressure, the distribution of existing antigenic polymorphisms affects the population structure in which newly selected mutants spread and may provide insight to the evolution and selection of parasite populations over time [10]. Polymorphisms of *PvDBPII *require further exploration among vivax isolates from different geographic areas, particularly south-east Asia where a large proportion of vivax infections take place. Therefore, the gene polymorphisms within the region II domain of the *P. vivax DBP *among Thai *P. vivax *isolates were investigated.

## Methods

### *P. vivax *samples and DNA preparation

*Plasmodium vivax *isolates were obtained from malaria patients with informed consent at the outpatient clinic, Hospital for Tropical Diseases, Faculty of Tropical Medicine, Mahidol University, Bangkok, from May, 2002 to June, 2003 under a protocol approval by the Ethics Committee, Faculty of Tropical Medicine, Mahidol University. The confirmation of *P. vivax *infection was done by microscopic examination of the parasites in thick and thin blood smears. Approximately 1 ml of venous blood was collected from each individual in 0.5 M ethylene-diamine tetra-acetic acid (EDTA) tubes and kept at -20°C until use. Genomic DNA was extracted from 200 μl of blood using a High Pure PCR Template Preparation Kit (Roche, Mannhein, Germany) according to the manufacturer's protocol.

### Genotyping of *P. vivax *isolates at the *PvDBPII *locus

*P. vivax DBPII *gene was amplified by PCR using the oligonucleotide primers, DBPII-F; 5'-CACCACGATCTCTAGTGCTATTATA-3' and DBPII-R; 5'-TGTCACAACTTCCTGAGTATT-3' [11]. PCR cycling conditions were 94°C for 3 min, 55°C for 1 min, and 72°C for 1 min for 35 cycles followed by a 10 min extension at 72°C. PCR products were stained with 0.1 μg/ml ethidium bromide and visualized by digital photography under ultraviolet light. The PCR products were run adjacently to 0.1 μg/lane of a 2-log DNA ladder (NewEngland BioLabs, Inc. MA, USA) as a standard DNA marker.

### Purification of PCR products, DNA cloning and DNA sequencing of *PvDBPII *gene

PCR products were purified by QIAquick PCR purification Kit (Qiagen Inc., Valencia, CA) following the manufacturer's instructions. The PCR products were cloned directly into pCR-Blunt II-TOPO cloning vector using a Zero Blunt TOPO PCR cloning kit (Invitrogen, La Jolla, CA). For DNA sequencing of the species-specific amplicons, excess dNTPs and unincorporated primers were eliminated using the QIAquick PCR purification kit (Qiagen Inc., Valencia, CA). DNA sequencing was performed by fluorescence based methodologies using MegaBACE DNA Analysis Systems (Amersham Biosciences, Piscataway, NJ). The sequences have been submitted to the GenBank under the accession number EF219451, EF368159–368180, EF379127–379135. Due to the possibility of multiple genotypes within parasites taken from one individual, direct sequencing was not performed. Instead, at least five clones were sequenced from each corresponding PCR product and sequences of these had to be identical to meet our quality control standards.

### Analysis of *PvDBPII *gene sequences

The alignment of complete sequences of 30 *PvDBPII *genes was analysed by Sequencher ver 4.2 software (Gene Code Corporation, Ann Arbor, MI) [12,13]. Sequences were aligned using CLUSTALX Multiple Sequence Alignment Program developed by the National Center for Biotechnology Information (NCBI, Bethesda, MD). The percent similarity was assessed using BLASTN (NCBI, Bethesda, MD) and BioEdit ver 7.0 software (Tom Hall Isis Therapeutics, Isis Pharmaceuticals, CA), DnaSP ver 4.0 [14] and MEGA ver 4.0 Beta program [15].

### Analysis of natural selection

Evidence of positive natural selection was determined by comparing the rate of nonsynonymous and synonymous substitutions in region II of the *P. vivax DBP *gene. The rates of substitutions were estimated using the Nei and Gojobori's method [16] with the Jukes and Cantor correction [17,18] as implemented in the MEGA program. Standard error was determined by 1000 bootstrap replications. Negative selection acting on most coding genes can be identified typically when the rate on nonsynonymous mutations is less than the rate of synonymous mutations (*k*_n _<*K*_s_). However, when positive selection is acting on a gene, the rate of nonsynonymous mutations will exceed that of synonymous mutations (*k*_n _> *K*_s_). Two other tests of neutrality were performed by Tajima's D test, Fu and Li's *D- *and *F*-tests on DnaSP 4 software using *P. knowlesi *DBP as an outgroup. Tajima's D test compares the estimation of nucleotide diversity calculated in two ways (θ calculated from the number of segregating sites and π calculated from average pairwise nucleotide diversity) in order to test for a departure from neutrality. Fu and Li's *D *and *F *test, departures from neutrality are identified as a deviation between estimates of θ (derived from the number of mutations in external branches of the phylogeny and from the total number of mutations giving the index *D *or from the average pairwise diversity π giving the index *F*) [19].

### Phylogenetic analysis

Phylogenetic analysis was used to investigate the associations of *PvDBPII *gene with sequences elucidated from different geographical regions. The gene tree was constructed using regions common to all available *PvDBPII *sequences. Forty five *PvDBPII *genes sequences found in GenBank were compiled and compared to Thai isolates including the sequences from a reference strain, Sal I, one individual sequence from Vietnam, Indonesia, Brazil and India as well as 2, 3, 3 and 31 sequences from Bangladesh, Colombia (COL), South Korea (SK) and Papua New Guinea (PNG), respectively (Table 1). A phylogenetic tree was derived from the aligned nucleotide sequences using the Neighbour-Joining (NJ) method with 1000 bootstrap replicates, the Tamura's three-parameter distance model as implemented in the MEGA ver 4.0 Beta program [20].

**Table 1:** *PvDBPII *sequences deposited in GenBank used in the study

species	locality	GenBank accession number
*P. vivax*	Papua New Guinea	AF289480–289653, AF469522–469602, AY970848–970925, AF291096, AF695565, DQ156519
*P. vivax*	Colombia	AY341907, DQ156513, AY341899
*P. vivax*	Korea	AF215737, AF215738, DQ156523
*P. vivax*	India	DQ156514
*P. vivax*	Brazil	DQ153520
*P. vivax*	Vietnam	DQ156518
*P. vivax*	Indonesia	DQ156521
*P. vivax*	Sal I	M37514, DQ156512

## Results

### PCR products and genetic polymorphisms of *PvDBPII *gene among Thai isolates

**Figure 1:**
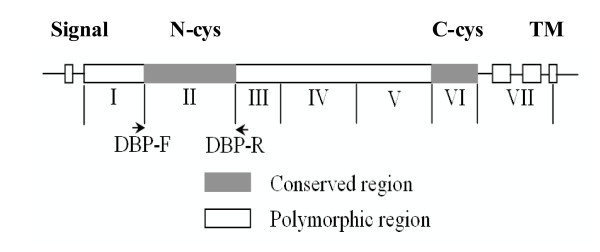
Schematic diagram of *PvDBPII *gene showing the location of the primers.

The PCR amplification of the *P. vivax DBPII *gene from 30 Thai isolates yielded DNA fragments of approximately 990 base pairs in length (Figure 1). A 900 bp was selected after excluding primer regions and adjacent bases which did not give uniformly reliable sequences. DNA analysis in comparison to the *PvDBP *gene of Sal I reference strain revealed amino acid polymorphisms across the entire domain II of *PvDBP *protein (300 amino acids) among Thai isolates. Among the 30 polymorphic sites, 29 showed monomorphic mutation (changed into one amino acid-type) and only a position 386(N/Q) showed dimorphic mutation (changed into two amino acid-types) (Table 2). Among 300 amino acids within *PvDBPII*, 25 nonsynonymous (representing 83% of all isolates) and five synonymous mutations were identified (Table 2). High frequencies of variant amino acids (>50%) were found and included L424I (86%), D384G (76%), W437R (63%), R390H (56%), and I503K (56%) residue polymorphisms.

**Table 2:** Amino acid changes found in *PvDBPII *gene among 30 Thai isolates, comparing to *DBP *Sal I sequence.

Position of the amino acid												▼		▽	▼
	268	276	281	306	308	333	351	367	371	375	378	384	385	386	390
SalI	CGT	AAC	GTT	TTT	AGG	CTT	AGT	ATC	AAA	AAT	CGC	GAT	GAA	AAG	CGT
Thai	AGT	AAT	GTA	TTG	AGT	TTT	TGT	ACC	GAA	GAT	CGT	GGT	AAA	AAT/CAG	CAT
SalI	R	N	V	F	R	L	S	I	K	N	R	D	E	K	R
Thai	S	N	V	L	S	F	C	T	E	D	R	G	K	N/Q	H
a	(1/30)	(1/30)	(1/30)	(2/30)	(8/30)	(14/30)	(1/30)	(2/30)	(6/30)	(7/30)	(30/30)	(23/30)	(14/30)	(13/30)	(17/30)
b	3	3	3	6	26	46	3	6	20	23	100	76	46	43	56

Position of the amino acid					▼			▼					▼		

	398	404	417	419	424	433	436	437	447	464	475	486	503	507	513
Sal I	TCT	ACA	AAT	ATA	TTA	CAG	AGA	TGG	TCA	ATC	CCA	CAA	ATA	AAC	ACG
Thai	ACT	AGA	AAA	ATG	ATA	AAG	ACA	CGG	TCC	ATA	GCA	GAA	AAA	CAC	AAG
Sal I	S	T	N	I	L	Q	R	W	S	I	P	Q	I	N	T
Thai	T	R	K	M	I	K	T	R	S	I	A	E	K	H	K
a	(3/30)	(3/30)	(11/30)	(1/30)	(26/30)	(1/30)	(1/30)	(19/30)	(1/30)	(1/30)	(3/30)	(2/30)	(17/30)	(1/30)	(1/30)
b	10	10	36	3	86	3	3	63	3	3	10	6	56	3	3

a Showing the detection frequency of each amino acids variant on *PvDBPII *protein among 30 Thai isolates.

b Percentage of detection frequency calculated from value in panel "a".

▼ High prevalence (>50%) of variant amino acids.

▽ Dimorphic mutation (changed into two amino acid-types)

**Figure 2:**
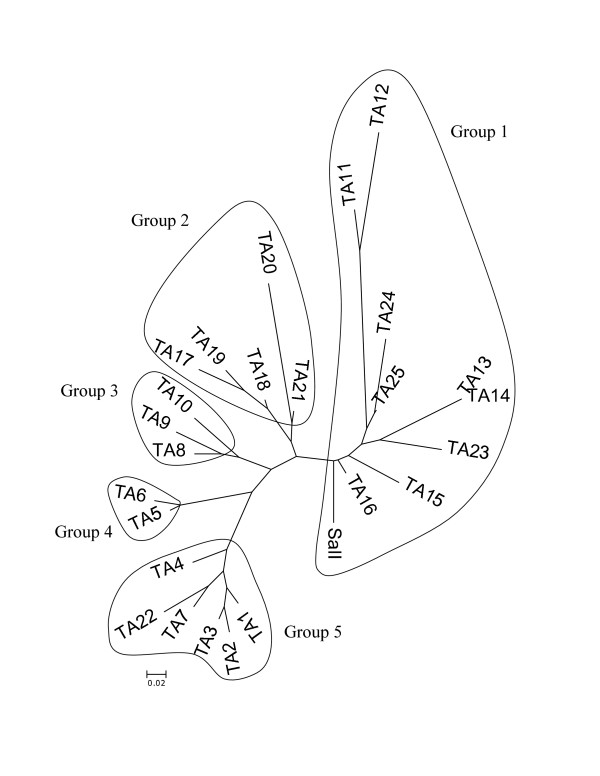
Neighbour-Joining tree of *PvDBPII *amino acid sequence of 25 variants observed among 30 Thai isolates. The *PvDBPII *haplotype for each variant could be seen in Table 3.

Based on the variation of nonsynonymous mutations and in comparison to Sal I the Thai haplotyes were designated as TA (TA1-TA25). TA1, TA5 and TA11 haplotypes were identified in two isolates while TA17 was identified in three isolates. The other 21 haplotypes were identified in single isolates (Table 3). The cluster analysis of *PvDBPII *amino acid sequences among 25 haplotypes based on Neighbour-Joining method was organized into five main groups (Figure 2).

**Table 3:** *PvDBPII *haplotypes observed among 30 Thai isolates.

Haplotype name	Amino acid haplotype*	No. of isolate observed (%)
TA 1	RFRFSIKDGKNHSTKIIQRRPQKNT	2(6.6)
TA 2	RFSFSIKDGKNHSTKIIQRRPQKNT	1(3.3)
TA3	RFSFSIKDGKKHSTKIIQRRPQKNT	1(3.3)
TA 4	RFRLSIKDGKNHSTKIIQRRPQKNT	1(3.3)
TA 5	RFRFSIENGKNRSTNIIQRRPQINT	2(6.6)
TA6	RFRFSIENGKNRSTKIIQRRPQINT	1(3.3)
TA 7	RFSFSIKDGKNHSTNIIQRRPQINT	1(3.3)
TA 8	RFRLSIENGEKHSTKIIQRRPQKNT	1(3.3)
TA 9	RFRLSIENGEQRSTKIIQRRPQKNT	1(3.3)
TA 10	RFRLSIENGEKRSTKMIQRRPQINT	1(3.3)
TA 11	RLSLLSIKNGKNHTRNIIQRWPQINT	2(6.6)
TA 12	SFSLSIKNGKNHTRNIQRWPEINT	1(3.3)
TA 13	RFRLSTKNGEKHSTNIIQRWPQKHT	1(3.3)
TA 14	RFRLSTKNGEKHSTNIIQRWPQKHT	1(3.3)
TA 15	RFSLSIKNGEKRSTNIIQRWPQKNT	1(3.3)
TA 16	RFRLSIKNGEKRSTNIIQRWPQINT	1(3.3)
TA 17	RFRFSIKNDEKRSTNIIQRRAQKNT	3(10)
TA 18	RFRLSIKNDEKRSTNIIQRRPQKNT	1(3.3)
TA19	RFRFSIKNDKKRSTNIIQRRPQKNT	1(3.3)
TA 20	RFRLSIKNDKKRSTKIIKTRPQINT	1(3.3)
TA 21	RFRLSIKNDKKRSTNIIQRWPQINT	1(3.3)
TA 22	RFSFSIKDGKNHSTNILQRRPQKNT	1(3.3)
TA23	RFRLSIKNGEKHSTNILQRWPQKNK	1(3.3)
TA 24	RFRLCIKNGEKHSTNILQRWPEINT	1(3.3)
TA 25	RFRLSIKNGEKHSTNILQRWPQINT	1(3.3)

* Amino acid residues included in the haplotypes are as follows: 268, 306, 308, 333, 351, 367, 371, 375, 384, 385, 386, 390, 398, 404, 417, 419, 424, 433, 436, 437, 475, 486, 503, 507, 513.

### Evidence of natural selection

To investigate whether natural selection contributed to generation of this diversity, the ratio of nonsynonymous (*K*_n_) to synonymous substitutions (*K*_s_) was compared between Thai and Sal I isolates. The number of nonsynonymous substitutions per nonsynonymous sites (*K*_n_) (0.00956) exceeded the number of synonymous substitutions per synonymous sites (*K*_s_) (0.00754). The *K*_n_/*K*_s _ratio was 1.270 predicting that positive selection may be occurring in region II of *DBP *favouring the fixation of amino acid replacement in certain areas of the protein. The statistic tests detecting departure from neutrality showed evidence that the observed polymorphism might be trending natural selection. Tajima's D value was -0.084549 with P > 0.10. The Fu and Li, *D *and *F *values were -0.92031 with P > 0.10 and -0.7654 with P > 0.10, respectively.

### Phylogenetic tree of *PvDBPII*

**Figure 3:**
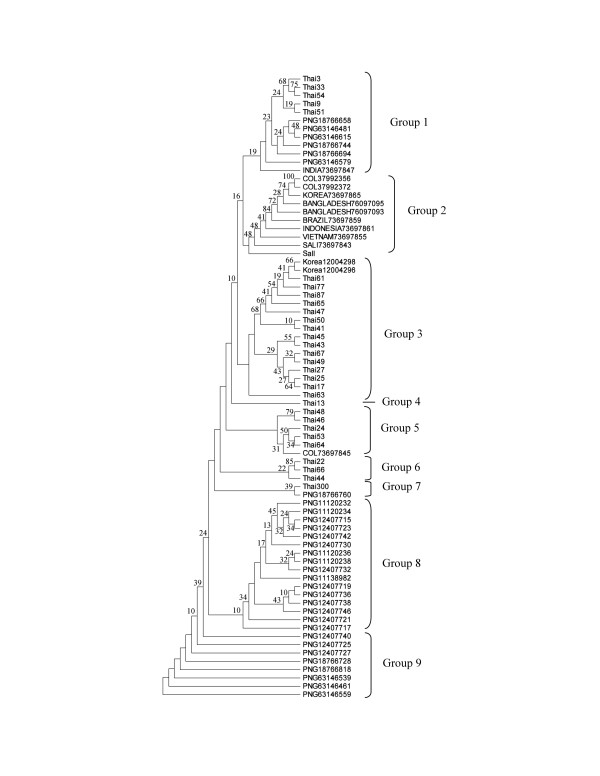
Phylogenetic tree of *PvDBPII *obtaining from Thai *P. vivax *isolates and other isolates from different geographical malaria endemic areas, *i.e*. PNG (Papua New Guinea), India, COL (Colombia), Korea, Bangladesh, Brazil, Indonesia, Vietnam, and Sal I. Numbers at nodes indicate percentage support of 1000 bootstrap replicates (bootstrap support values below 10 are not presented).

Phylogenetic tree analysis of DNA sequences derived from region II of the *P. vivax DBP *constructs based on the Neighbour-Joining method using Tamura's three-parameter distance is shown in Figure 3. Variation of DNA sequence was categorized into nine distinct alleles groups. Thai isolates were widely distributed amongst groups 1, 3, 5 and 7 also containing isolates from different geographical regions. *PvDBPII *DNA sequence of Thai isolates was related to isolates sets from Papua New Guinea and India (group 1), Korea (group 3), Colombia (group 5) and a second Papua New Guinea set (group 7). The Sal I reference strain used in this study was included in group 2 composed of isolates from Colombia, Korea, Bangladesh, Indonesia, and Vietnam, but bootstrap analysis demonstrated that the Thai isolates were distinct from this group. Most isolates from Papua New Guinea formed a distinctive group comprised of 8 and 9.

## Discussion

The process invasion into erythrocyte is essential for survival of *P. vivax *parasite and requires *DBP *to bind the Duffy blood group antigen that act as erythrocyte receptor, Duffy antigen receptor for chemokines (DARC). *Plasmodium vivax *utilizes genetic diversity of individual functional proteins to evade the host immune system and resist many anti-malarial drugs [21]. In this study, polymorphisms and natural selection was assessed and suggested positive natural selection in *PvDBPII *of Thai isolates. Although, non significant could be observed trending positive selection. As this is only a glimpse at the overall population, a wide range study including more subjects from different areas of Thailand would give a more detailed picture.

Analysis of 300 amino acids of *PvDBPII *protein relative to the Sal I reference strain, resulted in 25 nonsynonymous mutations being found. In addition, 142 nonsynonymous mutations were reported in 76 Papua New Guinea isolates [22]. The nonsynonymous mutations in *PvDBPII *might have an effect on parasite binding to erythrocyte receptors. Nonsynonymous mutations often times result in amino acid substitutions that alter charge and therefore host immune/antibody recognition [6]. Thus, excessive stable polymorphism generation within the *PvDBPII *ligand domain from high rate of nonsynonymous polymorphisms may promote the parasite escape of host immune response [9].

The high frequency (>50%) of L424I (86%), D384G (76%), W437R (63%), R390H (56%), and I503K (56%) residues were found in Thai isolates relative to Sal I *PvDBP *sequence. The finding is in contrast to previous studies showing R308S (67%), D384G (66%), and S447K (59%) in Papua New Guinea, D384G (59%) in Colombia and D384G (85%) in Brazil isolates [23]. Of the following variants N417K, W437R and I503K, all having been shown to be involved in evasion to antibody neutralization, variant N417K was found in low frequency (36%) while W437R and I503K were high (63% and 56% respectively) in Thai isolates. These results were different from what has been currently reported in that the low frequencies of variants N417K (27.5%) and W437R (27.5%) and the high frequency of variant I503K (55%) found in Brazil and low frequencies of all three variants (N417K, W437R, I503K) found in either Colombia (47%, 18%, 12%, respectively) and/or Papua New Guinea (23%, 26%, 29%, respectively) (Table 4) [23]. N417K, W437R and I503K have recently been shown to not be directly involved in erythrocyte binding, but possibly in inhibition of antibody binding to erythrocyte receptor [24]. Moreover, the variant amino acid T404R was identified in *PvDBPII *domains of three Thai isolates (Table 2) which is similar to the recent data in Papua New Guinea [22]. Other Thai variants, namely R308S, K371E, D384G, E385K, N386(N/Q), R390H, T404R, N417K, L424I, W437R, S447S and I503K, were also found in isolates of other regions, demonstrating commonality with a progenitor clone (Table 4). The other 18 polymorphic residues of *PvDBPII *ligand domain were recognized only among Thai isolates, indicative of divergence from a progenitor clone.

**Table 4:** Frequencies of the most common variant amino acids in *PvDBPII*, comparing to Sal I sequence (accession number M37519)

#	R308S	D384G	K386N	K386Q	N417K	L424I	W437R	S447K	I503K
Thai	26	76	40	3	36	86	63	0	56
COL*	0	59	23	0	47	47	18	0	12
PNG*	67	66	8	11	23	34	26	59	29
Brazil*	12.5	85	12.5	0	27.5	32.5	27.5	0	55

* The frequencies of the common variant amino acid data were referable according to Sousa et al. 2006.

# The first letter represented the amino acid in that position in Sal I sequences, and the other letter represents the substituted amino acid.

COL: Colombia, PNG: Papua New Guinea

The study showed that 25 *PvDBPII *haplotypes clustered into five main groups (dominant haplotypes) (Table 3) on the basis of nonsynonymous mutations is similar, but not entirely the same, with data from Papua New Guinea isolates whose 27 different *PvDBPII *haplotypes clustering into three dominant *PvDBPII *haplotypes. Potentially dominant *PvDBPII *haplotypes observed in these populations could be less immunogenic, thereby may help promoting parasite success and escaping the host immune response [22].

Phylogenetic analysis of *PvDBPII *suggested that Thai isolates fell into 6 different alleles groups. Among these, group 1 formed a group with a subset of Papua New Guinea isolates. Groups 4 and 6 were unique among Thai *P. vivax *isolates. However, the rest of the allele groups that Thai isolates fell into were more related to the isolates from Korea, India, and Colombia than from Papua New Guinea (Figure 3). The observations derived from the *PvDBPII *phylogeny suggested that single Indian isolate found in group 1 appeared to share a common ancestor close to a subset of Thai and Papua New Guinea isolates, demonstrating common ancestoral origins. Thai and Papua New Guinea isolate subsets in group 7 also may have shared common ancestor heritage. Recently, it was reported that travellers' malaria among foreigners at the Hospital for Tropical Diseases, Bangkok, Thailand including India and Papua New Guinea patients were though to have acquired their infections in their countries. Malaria importations from country to country can occur by either immigration or travel, and changing malaria attack rates in the countries of exposure are likely to influence the incidence of imported disease [25]. Additionally, these results demonstrated that there is some difference in the nucleotide or amino acid variation in *PvDBPII *among isolates from Thai, Korea, India, Papua New Guinea and Colombia, but most importantly, unique variants from Thailand do exist and should be considered in future vaccine development of *PvDBPII*. It should be noted that the present highly diverse phylogenetic tree was constructed relying on polymorphisms within a single gene, demonstrating the high degree of topological variation that can be found using this technique, but also highlighting the problems that can be associated with gene sampling in phylogenetic studies [26]. Use of a combination of several genes with different rates of evolutions has been suggested as an efficient way to overcome this difficulty to prove the existence of a unique tree relating these sequences [27]. Finally, it must also be pointed out that in many situations a single-gene phylogeny may be interesting in itself. Awareness of the problems of orthology (genes in different species that derive from a common ancestor) assignment and tree reconstruction artifacts should be considered.

## Conclusion

The results indicated that *PvDBPII *gene among Thai isolates is genetically diverse. Nonsynonymous polymorphism in *PvDBPII *was the predominant result of which variant L424I showed the highest frequency (86%). Five dominant *PvDBPII *haplotypes could be clustered among Thai isolates. The high frequency of polymorphisms and the presence of distinct alleles within *PvDBPII *gene among *P. vivax *from different geographical areas could provide complication in malaria vaccine development, but should be considered important parts of the development process.

## List of abbreviations used

*PvDBPII*: Domain II of *Plasmodium vivax DBP *gene; DARC: Duffy antigen receptor for chemokines

## Authors' contributions

SRM and SK designed the study, SK, MMF, SRM, KES, DEL and TK, were responsible for the supervision of the work, KES, TK, DEL, and PG were responsible for the sequences and phylogenetic analysis, SK, MMF, SRM, KES, DEL, TK, and PG drafted the manuscript. All authors read and approved the final manuscript.
